# Initial Validation of the Psychobiosocial States in Physical Education (PBS-SPE) Scale

**DOI:** 10.3389/fpsyg.2018.02446

**Published:** 2018-12-06

**Authors:** Laura Bortoli, Francesca Vitali, Rossana Di Battista, Montse C. Ruiz, Claudio Robazza

**Affiliations:** ^1^BIND–Behavioral Imaging and Neural Dynamics Center, Department of Medicine and Aging Sciences, University “G. D'Annunzio” of Chieti-Pescara, Chieti, Italy; ^2^Department of Neurosciences, Biomedicine, and Movement, University of Verona, Verona, Italy; ^3^Department of Medicine and Aging Sciences, University “G. D'Annunzio” of Chieti-Pescara, Chieti, Italy; ^4^Faculty of Sport and Health Sciences, University of Jyväskylä, Jyväskylä, Finland

**Keywords:** emotion, feelings, psychobiosocial states, IZOF model, physical education, assessment

## Abstract

The purpose of this cross-sectional investigation was to examine the item characteristics, factor structure, reliability, convergent validity, and nomological validity of the Psychobiosocial States in Physical Education (PBS-SPE) scale. In Study 1, a sample of 1,030 students (582 girls and 448 boys, 10 to 19-year-olds), drawn from middle or high schools, rated the intensity of the 20 items version of the PBS-SPE scale thinking about the feelings they had usually experienced in physical education classes. In Study 2, an additional sample of 1,025 students (578 girls, 447 boys, 10 to 19-year-olds), rated the 16 items of the final version of the scale. Two subsamples also completed an affective-related measure (i.e., the Physical Activity Enjoyment Scale) and two motivation scales often used in the physical education domain (i.e., the Teacher-Initiated Motivational Climate in Physical Education Questionnaire and the Situational Motivation Scale). Exploratory structural equation modeling and confirmatory factor analyses of the data showed that a two-factor, 16-item solution (i.e., 8 pleasant/functional items and 8 unpleasant/dysfunctional items) of the PBS-SPE scale reached satisfactory fit indices. Multi-group comparisons provided support for measurement and structural invariance across samples, gender, and age. Convergent and nomological validity was also upheld. Overall, the findings offer support for the use of a new instrument in the assessment of PBS-SPE settings.

## Introduction

Emotions are frequent, pervasive, manifold, and substantially related to motivation, learning, performance, and well-being in the educational domain and other achievement endeavors (Pekrun and Linnenbrink-Garcia, 2014). Pleasant emotions positively influence self-regulatory motivational and cognitive processes, such as creativity, flexible thinking, and holistic problem-solving, whereas unpleasant emotions determine disengagement, more analytical thinking, and inflexible information processing (Pekrun et al., 2009). In an integrative perspective, Pekrun's (2006) control-value theory conceptualizes emotions as a set of interrelated affective, cognitive, motivational, and physiological processes. Thrilling experiences, for example, can entail emotional (feeling excited), cognitive (being focused), motivational (being fascinated), and physiological activation (increased heart rate). In contrast, anxiety can involve feelings of distress, worry, withdrawal tendencies, and increased peripheral vascular resistance. Considerable research supports the contentions of the control-value theory (for a meta-analysis, see Huang, 2011).

In the sport setting, Hanin (2000, 2007, 2010) individual zones of optimal functioning (IZOF) model is a leading theoretical framework to the study of emotions that shares some similarities with the control-value theory (Pekrun, 2006). Indeed, the IZOF model incorporates, among others, a variety of affective, cognitive, motivational, and bodily-somatic components. Bortoli and colleagues have extensively applied the IZOF-based conceptualization of emotional states to the physical education context (e.g., Bortoli et al., 2014, 2015, 2017). As a holistic approach to the study of individual experiences, the IZOF model encompasses a wide range of psychobiosocial states related to performance. Psychobiosocial states include eight interactive modalities: (a) affective, cognitive, motivational, and volitional (psychological); (b) bodily-somatic and motor-behavioral (biological); and (c) operational and communicative (social). The affective modality can be perceived as pleasant or unpleasant (hedonic tone) and to have a functional or dysfunctional impact on performance, whereas all other non-emotion-related modalities can be perceived as functional or dysfunctional. Research findings uphold this conceptualization (for reviews, see Ruiz et al., 2017; Robazza and Ruiz, 2018).

Previous assessments of psychobiosocial states in sport and physical education settings have been mostly conducted through stimulus lists including pleasant/unpleasant and functional/dysfunctional items targeting the psychobiosocial modalities of the individual achievement experience (Ruiz et al., 2016; Di Battista et al., 2018). Each item representing a discrete state is comprised of three or more descriptors aimed to transmit a clear representation of an individual's experience in sport or physical education setting. Examples of functional and dysfunctional items for each modality, based on previous studies, are: “joyful” and “apprehensive” (affective modality); “attentive” and “distracted” (cognitive); “committed” and “uncommitted” (motivational); “determined” and “unwilling” (volitional); “energetic” and “tired” (bodily-somatic); “effortless-movement” and “uncoordinated movement” (motor-behavioral); “effective task-execution” and “inconsistent-task execution” (operational); “connected” and “disconnected” (communicative). In a trait-like assessment, participants are asked to think about their typical experiences in sport (Robazza et al., 2016) or physical education (Bortoli and Robazza, 2007) and rate the intensity of items thinking about how they usually feel.

Despite the considerable amount of research conducted on psychobiosocial states, only two studies examined the two-factor structure (i.e., pleasant/functional and unpleasant/dysfunctional) and reliability of a trait-like version measure (Robazza et al., 2016) and a state-like version measure (Ruiz et al., 2018) for use with athletes, while only one study provided initial evidence of the two-factor solution and reliability of a scale comprised of 14 items (7 pleasant and 7 unpleasant) in a large sample of 11 to 14-year-old physical education students (Bortoli and Robazza, 2007). In the Bortoli and Robazza's study, however, the volitional modality of psychobiosocial states was not included because it was conceptualized as a separate modality later (Hanin, 2010). Gender and age measurement invariance, convergent validity, and nomological validity of the measure in physical education students remained unexplored. Research findings pointed out gender and age differences in physical activity levels, showing that physical activity declines during adolescence, and this decline is greater and begins earlier in girls than boys across ages 12–15 years (e.g., Nader et al., 2008; Dumith et al., 2011). These differences can be reflected in psychobiosocial states. For example, higher scores of pleasant/functional psychobiosocial states were observed in boys compared with girls (Bortoli et al., 2014; Di Battista et al., 2018). Notwithstanding the limitations in previous studies, pleasant/functional psychobiosocial states in physical education (PBS-SPE) have been consistently associated with a task-involving climate created by the teacher, high levels of self-determined motivation, and student intention to engage in long-term physical activity, whereas unpleasant/dysfunctional states have been related to an ego-involving climate and low levels of self-determined motivation (Bortoli et al., 2014, 2015, 2017; Di Battista et al., 2018). Therefore, research supports the need for a valid and reliable instrument to assess PBS-SPE, which can answer important research questions and have practical implications.

To address this need, in Study 1 we assessed item characteristics, factor structure, and reliability of an initial measure called the PBS-SPE scale. In Study 2, we cross validated the scale in an independent sample and examined: (a) gender and age invariance, (b) convergent validity comparing the scale with an affective-related measure (i.e., an instrument that measures related constructs), and (c) nomological validity comparing the scale with two motivation scales often used in the physical education domain. The affective-related measure was meant to gauge feelings of enjoyment experienced during physical activity. Pleasant emotional experiences have been associated with involvement of youngsters in sport and exercise (Côté and Hancock, 2016). Accordingly, enjoyable physical education programs are expected to promote intrinsic motivation toward physical activity and the adoption of an active lifestyle (Wallhead and Buckworth, 2004). In support to the convergent validity of the PBS-SPE scale, we expected to find a positive relationship between pleasant/functional psychobiosocial states and pleasant feelings experienced in physical education, and a positive relationship between unpleasant/dysfunctional states and unpleasant feelings.

Feelings of pleasure, excitement, interest, and fun are emphasized within both achievement goal theory (Nicholls, 1984; Roberts and Treasure, 2012) and self-determination theory (Deci and Ryan, 1985, 2000; Ryan and Deci, 2017), which are two leading motivational approaches extensively applied in school, exercise, and sport settings. Achievement goal theory conceptualizes dispositional goal orientation and perceptions of motivational climate as two interacting dimensions (i.e., individual vs. environment) that influence behavior. Two types of dispositional goal orientations, namely task and ego, were primarily examined in achievement goal theory (Nicholls, 1984). Task-oriented individuals tend to define success based on self-referenced criteria, such as personal improvement, progression in learning, and mastery of a task. In contrast, ego-oriented individuals evaluate their own success as a function of social comparison and external criteria, focusing on outperforming others and exhibiting high normative ability. Task-oriented physical education students were more intrinsically motivated and reported experiencing more pleasant activating emotions than their ego-oriented counterparts (Mouratidis et al., 2009).

Achievement goal theory also focuses on social environmental factors such as the motivational climate established by the teacher. Students perceive a task-involving climate in situations where personal improvement, effort, and learning are emphasized. In contrast, when the emphasis is placed on comparison and competitiveness among peers, the climate created is likely to be viewed as ego-involving (Ames, 1992). The motivational climate influences the individual goal orientation and the adoption of a certain goal of action and behavior (Roberts and Treasure, 2012). A task-involving climate was consistently positively linked to intrinsic motivation, enjoyment, and adaptive motivational processes (Liukkonen et al., 2010; Barkoukis and Hagger, 2013), whereas an ego-involving climate was shown to determine maladaptive consequences (Appleton and Duda, 2016).

Given the importance of achievement goal theory in the educational context, we used a measure of task-involving and ego-involving climate to test the nomological validity of the PBS-SPE scale. Nomological validity refers to the extent to which a scale relates to other constructs in a theoretically consistent manner. Both convergent validity and nomological validity are among the most widely accepted forms of construct validity (Hair et al., 2014). According to Raykov and Marcoulides (2011), construct validity “includes evidence in favor of assumptions that an instrument in question relates to other observed measures in such a way that is consistent with predictions derived on the basis of already-available theories and accumulated knowledge in a substantive area.” (p. 190). Using previous measures of psychobiosocial states, study findings in the physical education setting showed a positive relationship between pleasant/functional states and perceived task-involving climate, and between unpleasant/dysfunctional states and ego-involving climate (Bortoli et al., 2014, 2015, 2017). Thus, consistent with such findings we expected pleasant/functional states assessed with the PBS-SPE scale to be positively related to a task-involving climate, and unpleasant/dysfunctional states to be positively related to an ego-involving climate.

Nomological validity of the PBS-SPE scale was also examined using a measure developed within the framework of self-determination theory (Deci and Ryan, 1985, 2000; Ryan and Deci, 2017). Self-determination theory “…is centrally concerned with the social conditions that facilitate or hinder human flourishing. The theory examines how biological, social, and cultural conditions either enhance or undermine the inherent human capacities for psychological growth, engagement, and wellness, both in general and in specific domains and endeavors” (Ryan and Deci, 2017, p. 3). The theory comprises six mini-theories developed to explain a set of motivationally based phenomena. Central in the organismic integration mini-theory of the self-determination theory is the conceptualization of different types of motivation along the autonomy–control continuum. Various types of motivation are represented as lying on this continuum that ranges from high to low levels of autonomous (self-determined) motivation. Intrinsic motivation, deriving from inherent enjoyment or interest in the task, and integrated regulation, associated with the attainment of benefits considered important and worthy, are placed in the upper part of the self-determination continuum, while external regulation, linked to external demands, possible rewards, or avoidance of negative consequences represents extrinsic forms of motivation. Amotivation reflecting a lack of motivation is placed on the lower part of the continuum. Research results indicate that an autonomy-supportive context in physical education promotes autonomous motivation, enjoyment, participation, effort, and persistence (Mouratidis et al., 2011; Standage et al., 2012; see Ryan and Deci, 2017). Study findings in physical education showed pleasant/functional states positively related to intrinsic motivation and identified regulation, and unpleasant/dysfunctional states positively linked to external regulation and amotivation (Bortoli et al., 2014, 2017). Thus, positive relationships were expected between pleasant/functional psychobiosocial states and more autonomous forms of self-determination, and between unpleasant/dysfunctional states and less autonomous forms of self-determination.

## Methods

### Study 1

In Study 1 we examined the item characteristics, the factor structure, and the reliability of the PBS-SPE scale.

#### Participants

The initial sample consisted of 1,030 students (582 girls, 448 boys), aged 10–19 years (*M* = 15.68, *SD* = 2.50), from 10 middle or high schools in Central Italy. Students engaged twice a week in physical education classes throughout the academic year.

#### Measure

The 20-item scale used in this study derived from the English version of the Individualized Profiling of Psychobiosocial States (Ruiz et al., 2016). The PBS-SPE scale included 10 pleasant/unpleasant functional and 10 unpleasant/pleasant dysfunctional items arranged in rows of 80 adjectives (3–6 adjectives formed an item) gauging eight state modalities (i.e., affective, cognitive, motivational, volitional, bodily-somatic, motor-behavioral, operational, and communicative). The Affective modality was assessed by six rows of adjectives for pleasant/functional states(+), pleasant/dysfunctional states(–), functional anxiety(+), dysfunctional anxiety(–), functional anger(+), and dysfunctional anger(–). Two rows of synonym adjectives assessed functional or dysfunctional states representing the remaining seven modalities. Each psychobiosocial state modality was therefore indexed by an item, which included two or more synonym descriptors conveying a straightforward representation of an emotional related experience. Items were rated in intensity on a 5-point Likert scale ranging from 0 (*not at all*) to 4 (*very, very much*).

Confirmatory factor analysis (CFA) of data from a trait version of the scale assessing the experiences of Italian athletes revealed best fit indices for a two-factor, 15-item (8 functional, 7 dysfunctional) solution (Robazza et al., 2016). Factor analysis of a state version of the scale administered to Finnish athletes showed best fit indices for a two-factor, 14-item (7 functional, 7 dysfunctional) solution (Ruiz et al., 2018). These studies offered initial validity support for sport-specific tools in the assessment of psychobiosocial states.

#### Procedure

School headmasters, physical education teachers, and parents of minors were contacted and explained the general purpose of the study before asking students to participate in the investigation. Once permission was granted, the students and their parents signed an informed consent form in accordance with the Declaration of Helsinki. The university's ethics committee approved the study. Individual assessment was conducted at school, in groups of four or five students, without the presence of the teacher. The general purpose of the study was explained to the students at the beginning of each meeting. Emphasis was placed on confidentiality of individual results and the voluntary nature of participation. Participants were asked to complete the PBS-SPE scale thinking about their own feelings usually experienced in physical education classes. In particular, they were requested to select one descriptor from the first row of items that best reflected their experiences and then to rate it in intensity on the 5-point Likert scale. The selection and rating procedure was repeated for each of the 20 rows of items. The assessment took ~20–30 min.

#### Data Analysis

Data were initially screened for missing values, distribution, and multivariate outliers (Tabachnick and Fidell, 2013). Sixteen cases were removed from further analyses because of missing values or identified as outliers (Mahalanobis' distance, *p* < 0.001). On the final sample (*N* = 1,014), we computed the frequency of descriptors chosen for each modality and descriptive statistics. Pearson product-moment correlation coefficients, reliability alpha values, composite reliability values, reliability omega values, and average variance extracted of the latent variables were also calculated (Watkins, 2017).

To examine the internal structure of the PBS-SPE scale we conducted exploratory structural equation modeling (ESEM; Marsh et al., 2009; Morin and Maïano, 2011) that integrates exploratory and confirmatory factor analysis and in which all factor loadings and cross loadings are estimated (Asparouhov and Muthén, 2009). We also performed CFA, which is a more restrictive analysis than ESEM, where cross-loadings are constraint to zero. ESEM models were estimated using a Bi-Geomin orthogonal rotation for uncorrelated factors and the robust maximum likelihood estimator (MLR), while CFA models were estimated using the maximum likelihood parameter estimates (MLM) with standard errors and a mean-adjusted chi-square test statistic that is robust to non-normality (Byrne, 2012). We expected the two pleasant/functional and unpleasant/dysfunctional factors of the PBS-SPE scale to be correlated. When latent dimensions are not independent, at least three competing measurement models could represent the test structure (Canivez, 2016), namely, a first-order factor model, a higher order factor model, and a nested-factor model. Thus, these competing models were examined using CFA to account for the first-order correlated dimensions and to explore the hierarchical structure of the scale (see Brunner et al., 2012; Canivez, 2016). The higher-order model has paths specified from a second-order general factor—which we named “affect/functionality” on the PBS-SPE scale—to the first-order factors (i.e., pleasant/functional and unpleasant/dysfunctional) that in turn were linked to the observed indicators. In the higher-order model of the PBS-SPE scale, the influence of affect/functionality (the superordinate dimension) on observed indicators is fully mediated by the first-order factors. In contrast, in the nested-factor model (also named bifactor measurement model) both the general factor (i.e., affect/functionality) and the group factors (i.e., pleasant/functional and unpleasant/dysfunctional) had direct paths to the observed indicators. Thus, the direct influence of affect/functionality on the observed indicators is not mediated by pleasant/functional and unpleasant/dysfunctional factors.

We used different indices to assess model fit: chi-square (χ^2^), normed chi-square (χ^2^/*df*), comparative fit index (CFI), Tucker Lewis fit index (TLI), root mean square error of approximation (RMSEA), and standardized root mean square residual (SRMR). Values for χ^2^/df <5, CFI and TLI >0.90, and RMSEA and SRMR lower than 0.08, were considered reflective of acceptable fit (Browne and Cudeck, 1993; Schumacker and Lomax, 2004). Good fit was inferred when CFI and TLI values were close to.95, and RMSEA and SRMR were lower than 0.06 (Hu and Bentler, 1999). To compare the fit of alternative models, we also used the Akaike's Information Criterion (AIC) values and the parsimony comparative fit index (PCFI). All data analyses were conducted in M*plus* version 8.1 (Muthén and Muthén, 2017).

#### Results

All adjectives included in each item were selected by the students. The 10 most selected descriptors were: fierce [Anger(+), 66.76%], physically-charged [Bodily-somatic(+), 59.81%], attentive [Cognitive(+), 48.68%], sociable [Communicative(+), 48.54%], physically tired [Bodily-somatic(–), 47.15%], satisfied [Pleasant states(–), 43.12%], overjoyed [Pleasant states(–), 42.70%], focused [Cognitive(+), 42.42%], dissatisfied [Anxiety(+), 41.31%], and carefree [Pleasant states(+), 40.89%]. The 10 least chosen descriptors were: communicative [Communicative(+), 10.15%], aggressive [Anger(+), 9.04%], alert [Cognitive(+), 8.90%], vigorous [Bodily-somatic(+), 8.07%], joyful [Pleasant states(+) 7.79%], resentful [Anger(–), 7.23%], confident [Pleasant states(+), 6.95%], annoyed [Anger(–), 4.59%], complacent [Pleasant states(–), 3.34%], and purposeful [Volitional(+), 3.20%]. Descriptive statistics of items are presented in Table 1.

**Table 1:** Descriptive statistics for the sample of Study 1 and the sample of Study 2.

**Modality**	**Sample 1 (*****N*** = **1,014)**				**Sample 2 (*****N*** = **1,011)**			
	***M***	***SD***	**SK**	**K**	***M***	***SD***	**SK**	**K**
Pleasant states(+)	3.32	0.92	−0.18	−0.26	3.32	0.91	−0.24	−0.11
Anxiety(+)	1.56	0.82	1.53	1.99	1.56	0.81	1.53	2.08
Anger(+)	2.66	1.17	0.18	−0.86	2.69	1.18	0.22	−0.82
Cognitive(+)	3.12	0.84	−0.01	−0.06	3.10	0.87	−0.11	−0.05
Motivational(+)	3.33	0.96	−0.25	−0.23	3.32	0.95	−0.14	−0.35
Volitional(+)	3.23	0.99	−0.14	−0.38	3.26	1.02	−0.17	−0.46
Bodily-somatic(+)	3.14	1.00	−0.06	−0.46	3.16	1.04	−0.14	−0.45
Motor-behavioral(+)	3.18	0.96	−0.09	−0.26	3.11	0.97	−0.28	−0.26
Operational(+)	3.15	0.90	−0.14	−0.01	3.11	0.95	−0.09	−0.26
Communicative(+)	3.52	0.97	−0.34	−0.31	3.55	0.96	−0.30	−0.25
Pleasant states(–)	3.35	0.95	−0.19	−0.33	3.32	0.99	−0.23	−0.35
Anxiety(–)	1.46	0.78	1.88	3.58	1.42	0.71	1.88	3.62
Anger(–)	1.36	0.71	2.28	5.36	1.34	0.70	2.41	6.22
Cognitive(–)	1.84	0.84	0.82	0.33	1.79	0.85	1.00	0.63
Motivational(–)	1.51	0.80	1.69	2.68	1.50	0.77	1.66	2.61
Volitional(–)	1.58	0.80	1.46	2.02	1.58	0.80	1.44	1.89
Bodily-somatic(–)	1.69	0.85	1.26	1.45	1.72	0.89	1.22	1.07
Motor-behavioral(–)	1.65	0.87	1.49	2.11	1.66	0.91	1.45	1.73
Operational(–)	1.57	0.81	1.47	1.79	1.54	0.76	1.48	2.12
Communicative(–)	1.38	0.77	2.20	4.31	1.34	0.72	2.48	6.42

*(+), Item categorized as functional; (–), item categorized as dysfunctional. M, mean; SD, standard deviation; SK, skewness; K, kurtosis*.

Two-factor models were initially examined through ESEM including the full 20-item scale and a scale containing 16 items, resulting from the exclusion of potentially problematic items, based on high cross-loadings (>0.30) on the hypothesized latent factors, a high value of modification indices (>20), or a low loading (<0.40) on the expected factor. The final 16-item, first-order factor solution was then assessed using CFA and compared against two competing hierarchical models (i.e., a higher order factor model and a nested-factor model). Factor analysis results are contained in Table 2. In line with the findings of previous research (Robazza et al., 2016; Ruiz et al., 2018), ESEM on the 20-item scale showed that the items of the Pleasant states(–) modality (i.e., overjoyed, complacent, pleased, satisfied) and the Anxiety(+) modality (i.e., nervous, restless, discontented, dissatisfied) loaded onto the pleasant/functional and unpleasant/dysfunctional factors, respectively. Therefore, these modalities were retained in the respective factors. The same analysis yielded high cross-loadings (>0.30) on Anger(+), Anger(–), Communicative(+), and Communicative(–) modalities. After having discarded these four modalities, the subsequent ESEM analysis on a 16-item scale provided better fit indices. The Satorra-Bentler scaled chi-square difference test (ΔS-B χ^2^ = 283.790, Δ*df* = 62, *p* < 0.001), the AIC values, and the PCFI suggested fit superiority of the 16-item scale compared to the 20-item scale. CFA on the 16-item scale also yielded acceptable fit indices.

**Table 2:** Fit indices for the two-factor (i.e., pleasant/functional and unpleasant/dysfunctional) models of the PBS-SPE Scale from Study 1 (*N* = 1,014) and Study 2 (*N* = 1,011).

**Model**	**χ^2^(*df*)**	**χ^2^/*df***	**CFI**	**TLI**	**RMSEA (90% CI)**	**SRMR**	**AIC**	**PCFI**
**Study 1**								
20 items (ESEM)	728.046 (151)	4.821	0.924	0.904	0.061 (0.057–0.066)	0.035	44184.235	0.483
16 items (ESEM)	444.685 (89)	4.996	0.942	0.922	0.063 (0.057–0.069)	0.032	35030.432	0.667
16 items, first-order factor model (CFA)	585.938 (103)	5.689	0.924	0.912	0.068 (0.063–0.073)	0.054	35158.196	0.440
16 items, first-order factor model (CFA)^a^	488.819 (101)	4.840	0.939	0.928	0.062 (0.056–0.067)	0.054	35053.119	0.665
16 items, higher order factor model (CFA)	1280.111 (105)	12.192	0.815	0.789	0.105 (0.100–0.110)	0.448	35938.497	0.365
16 items, nested-factor model (CFA)	985.998 (91)	10.835	0.859	0.815	0.098 (0.093–0.104)	0.440	35639.647	0.576
**Study 2**								
16 items (ESEM)	535.220 (89)	6.014	0.928	0.903	0.070 (0.065–0.076)	0.035	35044.304	0.657
16 items, first-order factor model (CFA)^a^	578.154 (101)	5.724	0.924	0.910	0.068 (0.063–0.074)	0.057	35069.385	0.467

ESEM, Exploratory Structural Equation Modeling; CFA, Confirmatory Factor Analysis; χ^2^(df), chi-square (degrees of freedom); CFI, comparative fit index; TLI, Tucker Lewis fit index; RMSEA, root mean square error of approximation; SRMR, standardized root mean square residual; AIC, Akaike's Information Criterion; PCFI, Parsimony comparative fit index.

a *Two residuals allowed to correlate on the pleasant/functional factor and two residuals on the unpleasant/dysfunctional factor*.

**Figure 1:**
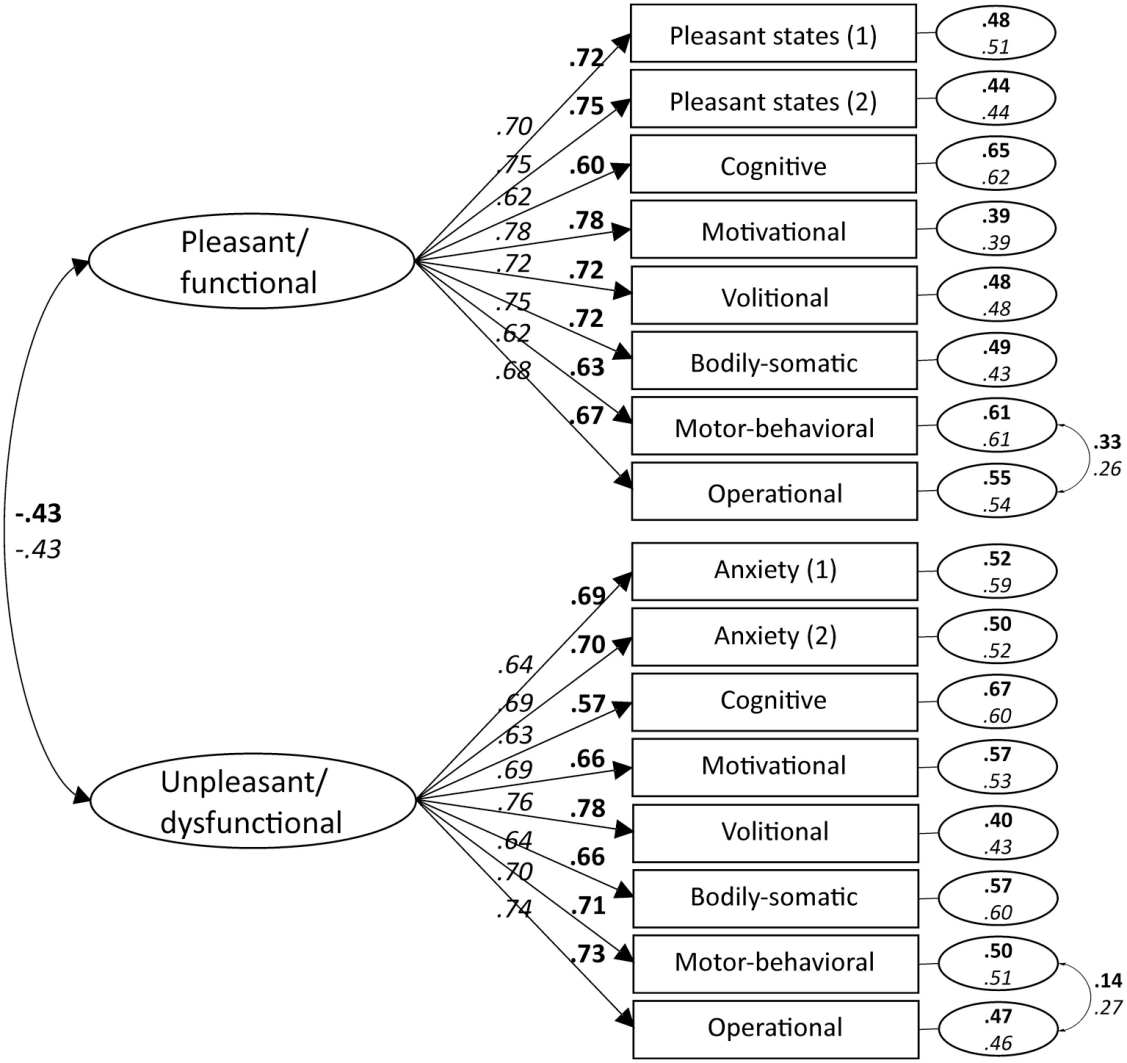
Standardized factor loadings, error variances, and correlations between latent constructs of the 16-item PBS-SPE scale derived from Study 1 (bold; *N* = 1,014) and Study 2 (italic; *N* = 1,011) based on confirmatory factor analyses. All factor loadings are significant at *p* < 0.001 (two-tailed).

Inspection of modification indices suggested correlating the errors of Motor-behavioral and Operational modalities in each factor. The Motor-behavioral modality refers to movement execution, while the Operational modality represents performance effectiveness. Given the content similarity between the two modalities, we judged sound to respecify the model allowing the correlation of residuals of Motor-behavioral(+) with Operational(+), and Motor-behavioral(–) with Operational(–). This change led to improved fit (ΔS-B χ^2^ = 100.370, Δ*df* = 2, *p* < 0.001). Finally, CFA fit indices resulting from both the higher order factor model and the nested-factor model did not fit the data well. In summary, both ESEM and CFA analyses supported a first-order factor model of the PBS-SPE scale, whereas hierarchical models were not supported. Figure 1 shows standardized factor loadings, error variances, and correlations between latent constructs (i.e., pleasant/functional and unpleasant/dysfunctional psychobiosocial states) of the 16-item PBS-SPE scale. Means, standard deviations, Cronbach's alpha values, composite reliability, reliability omega values, and average variance extracted for the latent variables are reported in Table 3.

**Table 3:** Sample size, means, standard deviations, Cronbach's alpha values (α), composite reliability (CR), omega values (ω), and average variance extracted (AVE) for the latent variables from Study 1 and Study 2.

**Sample**	**Latent variable**	**Girls**				**Boys**				**Total sample**			
		**Age**	***N***	***M***	***SD***	**Age**	***N***	***M***	***SD***	**α**	**CR**	**ω**	**AVE**
Sample 1	Pleasant/functional	10–13	100	2.80	0.70	10–13	79	3.30	0.64				
		14–15	146	3.12	0.73	14–15	96	3.50	0.61				
		16–17	201	3.13	0.70	16–17	122	3.46	0.62				
		18–19	135	3.16	0.69	18–19	135	3.43	0.67				
										0.887	0.887	0.736	0.496
	Unpleasant/dysfunctional	10–13	100	1.51	0.56	10–13	79	1.51	0.48				
		14–15	146	1.78	0.67	14–15	96	1.60	0.51				
		16–17	201	1.71	0.68	16–17	122	1.46	0.48				
		18–19	135	1.72	0.66	18–19	135	1.44	0.52				
										0.878	0.879	0.725	0.478
Sample 2	Pleasant/functional	10–13	98	2.81	0.69	10–13	79	3.32	0.67				
		14–15	146	3.12	0.75	14–15	102	3.46	0.64				
		16–17	199	3.14	0.71	16–17	119	3.45	0.66				
		18–19	135	3.09	0.73	18–19	133	3.40	0.69				
										0.889	0.889	0.739	0.502
	Unpleasant/dysfunctional	10–13	98	1.48	0.46	10–13	79	1.46	0.52				
		14–15	146	1.77	0.68	14–15	102	1.59	0.50				
		16–17	199	1.67	0.65	16–17	119	1.50	0.51				
		18–19	135	1.72	0.68	18–19	133	1.45	0.53				
										0.876	0.878	0.724	0.476

### Study 2

Study 2 aimed to cross validate in a second independent sample the two-factor, 16-item solution of the PBS-SPE scale observed in Study 1, and to examine measurement invariance across gender and age. An additional purpose of Study 2 was to explore convergent validity through correlations with an affective-related measure, and nomological validity in comparison with two motivation scales often used in physical education settings.

#### Participants

The demographic characteristics of the sample were similar to those of Study 1. The sample consisted of 1,025 students (578 girls, 447 boys), aged 10–19 years (*M* = 15.78, *SD* = 2.54), from 10 middle or high schools in Central Italy, taking part to physical education classes twice a week across the academic year. Procedures to obtain permissions from school headmasters, physical education teachers, and parents, as well as consent from students and approval from the university's ethics committee, were the same of Study 1.

#### Measures

We administered the two-factor, 16-item solution of the PBS-SPE scale drawn from the analysis conducted in Study 1 (see Appendix 1), the Physical Activity Enjoyment Scale (PACES; Kendzierski and DeCarlo, 1991), the Teacher-Initiated Motivational Climate in Physical Education Questionnaire (TIMCPEQ; Papaioannou, 1998), and the Situational Motivation Scale (SIMS; Guay et al., 2000).

The PACES is a 16-item scale to measure feelings of enjoyment associated with physical activity. The scale consists of 9 items loading onto a pleasant-feelings factor (e.g., “I enjoy it”) and 7 items loading onto an unpleasant-feelings factor (e.g., “I dislike it”). Students were asked to rate the items on a 5-point Likert scale ranging from 1 = *totally disagree* to 5 = *totally agree*, in relation to the feelings they have experienced during physical education classes. The two-factor solution has received empirical support in a large sample of Italian girls and boys aged from 11 to 19 years (Carraro et al., 2008).

The TIMCPEQ is a 12-item questionnaire to assess the individual's perceptions of task-involving and ego-involving motivational climates. It consists of two 6-item scales measuring students' perceptions of their teacher's emphasis on skill mastery and effort (e.g., “The physical education teacher is most satisfied when every student learns something new”), and the teacher's emphasis on social comparison and competition (e.g., “Only the students with the best records are rewarded”). Students were asked to rate the items on a 5-point scale, ranging from 1 = *strongly disagree* to 5 = *strongly agree*, thinking about the climate their teachers create in physical education classes. CFA of a version of the scale translated and adapted into the Italian language provided support for the two-dimensional structure of the questionnaire (Bortoli et al., 2008, 2017).

The SIMS is a 16-item scale to gauge the constructs of intrinsic motivation, identified regulation, external regulation, and amotivation. The scale was developed within the framework of self-determination theory, and in particular within the organismic integration mini-theory in which different types of motivation are classified along the autonomy–control continuum (Ryan and Deci, 2017). The SIMS comprises four 4-item scales reflecting the theorized constructs (e.g., intrinsic motivation, “I think that this activity is pleasant;” identified regulation, “I believe that this activity is important for me;” external regulation, “It is something that I have to do;” amotivation, “I do this activity but I am not sure if it is worth it”). Students were asked to rate the items on a 7-point scale ranging from 1 = *does not correspond at all* to 7 = *corresponds exactly*, thinking about their reasons for current engagement in physical education classes. CFA of the Italian version supported the four-dimensional structure of the scale (Bortoli et al., 2017).

#### Procedure

The PBS-SPE scale was administered following the procedure described in Study 1. A subsample of students (*n* = 223), aged 14–19 years, was also asked to fill out the PACES, while another subsample of participants (*n* = 751) of the same age was requested to complete the TIMCPEQ and SIMS.

#### Data Analysis

The initial screening of the data led to the removal of 14 cases due to missing values or because identified as multivariate outliers. Descriptive statistics, correlation coefficients, and reliability values of the study variables were computed on the final sample (*N* = 1,011). ESEM and CFA were then performed to examine the factorial validity of the 16-item PBS-SPE scale derived from Study 1.

Scale invariance across the two study samples was assessed through multigroup CFAs increasing parameter constraints one at a time (Byrne, 2012; Wang and Wang, 2012). For this purpose, we established a configural model as the baseline against which the subsequently specified models were compared. Then, increasingly stringent models were employed to test measurement and structural invariance (Farmer and Farmer, 2014). Measurement invariance testing entailed configural (i.e., same number of factors and factor loading pattern across groups), weak metric (i.e., equality of factor loadings), strong metric (i.e., equality of factor loadings and intercepts), and strict metric (i.e., equality of error variance and covariance). Structural invariance testing involved factor variance (i.e., equality of variance of factor scores), factor covariance (i.e., equality of covariance of factor scores), and factor mean (i.e., equality of latent means). Model comparisons were executed at each step using the Satorra-Bentler scaled chi-square difference (ΔS-B χ^2^). Furthermore, a difference in CFI between nested models ≤0.01 was considered a criterion of invariance (Cheung and Rensvold, 2002). Differences in RMSEA and SRMR values were also considered in the comparison of nested models (Chen, 2007).

Invariance across gender and age categories (i.e., 10–13, 14–15, 16–17, and 18–19 years) was examined using multiple indicator, multiple cause (MIMIC) model. In a MIMIC model, also called CFA with covariates (Brown, 2015), multiple indicators reflect the underlying latent factors, and the multiple causes (i.e., observed predictors) influence latent factors. The covariates in our models were gender, age, and their interaction. The application of MIMIC modeling was deemed more feasible than the use of multi-group CFA because the sample size was relatively unbalanced (e.g., a smaller number of boys and youngsters in our sample compared to girls and older students). In a MIMIC model, the effects of covariates on both latent factors and endogenous indicator variables enable to examine the effects of covariates on factors, as well as the effects of covariates on items. It is therefore possible to determine whether factor means and/or responses to the same items are invariant or not between groups controlling for the underlying factor. Gender and age covariates were dummy coded to represent group membership. A gender by age interaction term was also derived. The latent variables and indicators were then regressed onto the covariates and their interaction term.

Finally, convergent and nomological validity of the PBS-SPE scale were examined after having ascertained the factorial structure of the PACES, TIMCPEQ, and SIMS. Specifically, convergent validity of the PBS-SPE scale was assessed in comparison with the PACES (i.e., the degree to which the two scales were correlated), while nomological validity was established in comparison with the TIMCPEQ and SIMS.

#### Results

Descriptive statistics of study variables are contained in Table 1. As the table shows, higher values were reported for most functional items with lowest values reported for Anger(–) in both samples. ESEM and CFA results for the PBS-SPE scale are reported in Table 2. As it can be seen, ESEM and CFA fit indices on the 16-item PBS-SPE scale were acceptable, thus confirming the tenability of the solution obtained in Study 1. All indicators loaded in the expected factors with all factor loadings being significant and higher than 0.53 (see Figure 1). As Table 3 shows, higher values were reported for items in the functional latent factors across groups in both Study 1 and Study 2. Reliability scores as indicated by Cronbach' alpha, composite reliability, and omega values were all acceptable (>0.724). Values close to 0.500 of average variance extracted suggest adequate convergence of items (Hair et al., 2014), which in the current study are indicators of pleasant/functional and unpleasant/dysfunctional latent variables. Discriminant validity, namely, the extent to which the pleasant/functional factor is distinct from the unpleasant/dysfunctional factor (Hair et al., 2014) was also supported. Indeed, average variance extracted values for the two constructs were greater than the squared correlation estimate between the two constructs (*r*^2^ = 0.185).

Table 4 contains the results of multi-group comparisons assessing the invariance of the scale across the two study samples. As it can be seen, the CFA configural model fitted the data adequately, thus supporting the same factor structure (i.e., the same number of factors and the same patterns of free and fixed factor loadings) of the PBS-SPE scale across the two study groups. Full measurement and structural invariance of the scale was also found. This was indicated by the non-significant ΔS-B χ^2^ tests between the configural and all other nested models, and the <0.01 CFI value difference between the configural and other models.

**Table 4:** Fit indices for multi-group confirmatory factor analyses of the PBS-SPE scale.

**Independent variable**	**Model**	**χ^2^(*df*)**	**χ^2^/*df***	**CFI**	**TLI**	**RMSEA (90% CI)**	**SRMR**	**ΔS-B χ^2^ (Δ*df*)**	***p-*value**
Study group	Configural	1068.045 (202)	5.287	0.932	0.919	0.065 (0.061–0.069)	0.055		
	Weak metric	1081.614 (216)	5.007	0.932	0.924	0.063 (0.059–0.067)	0.057	15.129 (14)	0.369
	Strong metric	1104.974 (232)	4.763	0.931	0.929	0.061 (0.057–0.065)	0.057	31.018 (30)	0.415
	Strict metric	1102.739 (248)	4.447	0.932	0.935	0.058 (0.055–0.062)	0.058	49.819 (46)	0.324
	Factor variance	1101.326 (234)	4.707	0.931	0.930	0.061 (0.057–0.064)	0.058	30.982 (32)	0.518
	Factor covariance	1102.918 (233)	4.734	0.931	0.929	0.061 (0.057–0.064)	0.057	30.511 (31)	0.491
	Factor mean	1103.339 (230)	4.797	0.931	0.928	0.061 (0.058–0.065)	0.057	30.278 (28)	0.350
Gender and age	MIMIC	1149.219 (143)	8.036	0.926	0.912	0.059 (0.056–0.062)	0.050		

*χ^2^(df), chi-square (degree of freedom); χ^2^/df, chi-square/degree of freedom; CFI, comparative fit index; TLI, Tucker Lewis fit index; RMSEA, root mean square error of approximation; SRMR, standardized root mean square residual; ΔS-B χ^2^ (Δdf), Satorra-Bentler scaled chi-square difference test (degree of freedom difference). MIMIC analysis was conducted on the total sample (Study 1 and Study 2, N = 2,025)*.

Given the substantial invariance across groups, MIMIC analysis was conducted on the total sample (Study 1 and Study 2, *N* = 2,025; see main results in Table 4). The results showed acceptable fit of the model. The estimated parameters indicated significant effects of gender (0.514, *p* < 0.001) and age (0.085, *p* < 0.001) on pleasant/functional states, and an interaction effect of gender by age (−0.070, *p* = 0.009). Results also suggested a significant effect of age (0.046, *p* = 0.006) and an interaction effect of gender by age (−0.074, *p* = 0.001) on unpleasant/dysfunctional states. The effect of covariates on items was then examined to ascertain whether responses to the same items differed between groups. Significant effects were not observed and therefore item functioning did not differ across gender and age. Inspection of mean values of latent variables (Table 3) indicates lower levels of pleasant/functional states on 10 to 13-year-olds (boys and girls) than their older counterparts (age main effect), and a lower level of unpleasant/dysfunctional states on 10 to 13-year-old boys than older boys (gender by age interaction). Moreover, girls tended to experience lower levels of pleasant/functional states than boys, and higher levels of unpleasant/dysfunctional states (older girls in particular).

CFA results and reliability indices of the PACES, TIMCPEQ, and SIMS indicated acceptable factorial validity and reliability of the measures (see Table 5). The two-factor structure of the PACES was established after specification of correlated residual terms of six items on the pleasant subscale and two items on the unpleasant subscale (AIC with uncorrelated residuals vs. AIC with correlated residuals were 6446.065 and 6320.407, respectively). The average variance extracted for the two constructs was greater than the squared correlation estimate between the two constructs (*r*^2^ = 0.341), thereby supporting the discriminant validity of the measure. The two-factor structure of the TIMCPEQ was also confirmed after specification of correlated residual terms of two items in the task-involving climate subscale and two items in the ego-involving climate scale (AIC = 20516.238 vs. 20437.116). The squared correlation estimate between the two constructs (*r*^2^ = 0.021) was smaller than the average variance extracted. Finally, the four-factor structure of the SIMS was supported after specification of correlated residual terms of two items in the identified regulation subscale and other two in the external regulation subscale (AIC = 37543.333 vs. 37417.889). The squared correlation estimates among the four constructs (*r*^2^ ranging from 0.227 to 0.452) were smaller than the average variance extracted.

**Table 5:** Confirmatory factor analysis fit indices, Cronbach's alpha values (α), composite reliability *(*CR), omega values (ω), and average variance extracted (AVE) for the latent variables of the PACES (*N* = 223), the TIMCPEQ (*N* = 751), and the SIMS (*N* = 751) from Study 2.

**Instrument**	**Factors**	**χ^2^(*df*)**	**χ^2^/*df***	**CFI**	**TLI**	**RMSEA (90% CI)**	**SRMR**	**α**	**CR**	**ω**	**AVE**
PACES		209.411 (99)	2.115	0.932	0.918	0.071 (0.05–0.084)	0.057				
	Pleasant feelings							0.917	0.919	0.773	0.564
	Unpleasant feelings							0.816	0.810	0.669	0.406
TIMCPEQ		179.318 (51)	3.516	0.935	0.916	0.058 (0.49–0.067)	0.056				
	Task-involving climate							0.771	0.780	0.664	0.395
	Ego-involving climate							0.702	0.719	0.615	0.330
SIMS		401.742 (96)	4.185	0.946	0.933	0.065 (0.59–0.072)	0.048				
	Intrinsic motivation							0.894	0.827	0.743	0.682
	Identified regulation							0.802	0.869	0.824	0.512
	External regulation							0.812	0.832	0.778	0.546
	Amotivation							0.829	0.840	0.779	0.559

*χ^2^(df), chi-square (degrees of freedom); CFI, comparative fit index; TLI, Tucker Lewis fit index; RMSEA, root mean square error of approximation; SRMR, standardized root mean square residual*.

Latent factor correlations of the PBS-SPE scale with the convergent- and nomological-related measures are shown in Table 6. For the study purposes, we can note some relevant correlations in line with our expectations. According to Zhu's 2012 indications on how to judge correlation coefficients, moderate and moderately high positive correlations were found between the pleasant/functional factor of the PBS-SPE scale and pleasant feelings (PACES), task-involving climate (TIMCPEQ), intrinsic motivation (SIMS), and identified regulation (SIMS) factors. Negative correlations were shown between the pleasant/functional factor and unpleasant feelings (PACES), external regulation (SIMS), and amotivation (SIMS) factors. The unpleasant/dysfunctional factor of the PBS-SPE scale correlated positively with unpleasant feelings (PACES) and external regulation (SIMS) factors, and negatively with the intrinsic motivation (SIMS) factor. These results support both convergent and nomological validity of the PBS-SPE scale.

**Table 6:** Means, standard deviations, and Pearson-product moment correlation matrix for the latent variables from Study 2.

**Latent variables**	**(1)**	**(2)**	**(3)**	**(4)**	**(5)**	**(6)**	**(7)**	**(8)**	**(9)**	**(10)**
(1) PBS-SPE—Pleasant/functional	–									
(2) PBS-SPE—Unpleasant/dysfunctional	−0.362^a^, −0.459	–								
(3) PACES—Pleasant feelings	0.735^a^	−0.285^a^	–							
(4) PACES—Unpleasant feelings	−0.480^a^	0.584^a^	−0.584^a^	–						
(5) TIMCPEQ—Task-involving climate	0.393	-0.130			–					
(6) TIMCPEQ—Ego-involving climate	−0.078	0.157			−0.148	–				
(7) SIMS—Intrinsic motivation	0.663	−0.455			0.386	−0.123	–			
(8) SIMS—Identified regulation	0.479	−0.283			0.322	−0.078	0.651	–		
(9) SIMS—External regulation	−0.424	0.411			−0.287	0.182	−0.530	−0.476	–	
(10) SIMS—Amotivation	−0.442	0.367			−0.355	0.209	−0.490	−0.478	0.672	–
Mean (*N* = 223^a^, *N* = 751)	3.09^a^, 3.31	1.49^a^, 1.88	2.96	1.34	3.82	2.06	4.67	4.65	2.74	2.11
Standard deviation	0.70^a^, 0.68	0.53^a^, 0.66	0.73	0.42	0.55	0.57	1.26	1.23	1.38	1.12

a *First subsample. Data values without superscript are from the second subsample*.

We also derived summated scales by combining the variables measuring similar concepts into single variables. Specifically, we calculated an index of psychobiosocial states subtracting scores of unpleasant/dysfunctional states from scores of pleasant/functional states (Bortoli et al., 2017). Given that mean scores of students' pleasant/functional states were larger than scores of unpleasant/dysfunctional states, the higher the index score, the higher the level of pleasant/functional psychobiosocial states experienced. In a similar way, we computed an index of enjoyment and an index of motivational climate subtracting unpleasant-feelings from pleasant-feelings and ego-involving motivational climate from task-involving motivational climate, respectively. Finally, we computed a self-determination index using the mean scores of the subscales of the SIMS (Johnson et al., 2011). The following formula was applied: [self-determination index = +2(Intrinsic motivation) +1(Identified regulation) −1(External regulation) −2(Amotivation)]. Higher scores indicate higher levels of self-determined motivation. Positive correlations were observed between the psychobiosocial states index and all other indices (PACES, *r* = 0.718; TIMCPEQ, *r* = 0.292; SIMS, *r* = 0.653).

## Discussion

The purpose of the current study was to investigate the item characteristics, factor structure, reliability, gender and age invariance, convergent validity, and nomological validity of the PBS-SPE scale. Although extensive research has been conducted on psychobiosocial states in sport and physical education domains (see Ruiz et al., 2017), this is the first study to examine the validity of the most recent version of a tool designed to measure PBS-SPE. Study findings provided support to the factor structure and reliability of the PBS-SPE scale. The construct validity of the scale was also demonstrated in terms of convergent and nomological validity in comparison with other substantively related measures.

### Initial Validation

According to earlier research findings in sport (Robazza et al., 2016; Ruiz et al., 2018), all adjectives representing the eight modalities of a psychobiosocial state (i.e., affective, cognitive, motivational, volitional, bodily, motor-behavioral, operational, and communicative) were selected by the students. This result supports the relevance of the descriptors and the multimodality structure of a psychobiosocial state that encompasses both emotion and non-emotion content (Hanin and Stambulova, 2002; Ruiz and Hanin, 2004). Descriptive statistics (Table 1) showed mean intensity scores of items in the pleasant/functional subscale to be larger than scores of items in the unpleasant/dysfunctional subscale. This is consistent with the general aims of school physical education meant to promote student improvement and enjoyment through a pleasant and effective learning atmosphere (Bortoli et al., 2014, 2015, 2017).

In line with research findings on psychobiosocial states in sport (Robazza et al., 2016; Ruiz et al., 2018), factor analysis results yielded a two-factor solution of the scale. The final solution of the PBS-SPE scale was comprised of 16 items, 8 pleasant/functional and 8 unpleasant/dysfunctional. The items of the Pleasant states(–) modality (i.e., overjoyed, complacent, pleased, satisfied) loaded onto the pleasant/functional factor, while the items of the Anxiety(+) modality (i.e., nervous, restless, discontented, dissatisfied) loaded onto the unpleasant/dysfunctional factor. Thus, the hedonic valence of the two modalities tended to override the perceived functionality. In the IZOF framework (Hanin, 2000, 2007), the two items are deemed to reflect a pleasant/dysfunctional state [i.e., Pleasant states(–) modality] and an unpleasant/functional state [i.e., Anxiety(+)]. In the same view, emotion content is conceptualized based on the 2 × 2 interaction between hedonic valence (i.e., pleasant or unpleasant experience) and functionality (functional or dysfunctional impact on performance), resulting into four global emotion content categories: (a) pleasant-functional, (b) unpleasant-functional, (c) pleasant-dysfunctional, and (d) unpleasant-dysfunctional. It is argued that (a) pleasant-functional states (e.g., feeling confident and joyful) help the performer to generate and use the energy needed to execute a task, (b) unpleasant-functional states (e.g., feeling nervous and restless) have also energizing functions toward behavior, (c) pleasant-dysfunctional states (e.g., feeling overjoyed and satisfied) reflect a lack of energy or ineffective resource recruitment and use, and (d) unpleasant-dysfunctional states (e.g., feeling concerned and troubled) cause a waste of energy. In contrast to this theoretical classification, in the current investigation and the two studies on psychobiosocial scales in sport (Robazza et al., 2016; Ruiz et al., 2018) the Pleasant states(–) and the Anxiety(+) modalities were found to have opposite perceived effects than those expected. This result can be explained by the common and ingenuous belief that unpleasant states are always dysfunctional for performance and that pleasant states are always functional. Although research did not support this belief, a low level of individual's awareness of the subtle functional or dysfunctional effects of emotions on performance, irrespective to the hedonic valence, may have contributed to this finding. Furthermore, the typical view of an educational setting, properly focused on the process of learning, improving, and acquiring physical activity habits, can lead the hedonic valence of the physical education experience to override feelings associated with competitive outcomes.

ESEM analysis showed high cross-loadings for the anger modalities on functional and dysfunctional factors. Adjectives of functional anger (i.e., fighting spirit, fierce, aggressive) and dysfunctional anger (i.e., furious, resentful, irritated, annoyed) are most likely more appropriate in sport rather than in a physical education setting. Indeed, anger, aggression, and related feelings can occur quite frequently during competition in many sports, particularly in contact sports such as rugby and karate, not only as a reaction of frustrating events, but also as a means of energizing behavior for competitive achievements (Robazza and Bortoli, 2007; Ruiz and Hanin, 2011; Campo et al., 2012). ESEM also yielded high cross-loadings for the communicative modalities. We may speculate that both “communicative, outgoing, sociable, connected” and “uncommunicative, withdrawn, alone, disconnected” lists of adjectives, which entail interpersonal skills, can be individually considered as reflecting functional or dysfunctional social interactions, depending on a personal appraisal of the physical education and task requirements. Some students can be more or less communicative than others and prone to seek support from the teacher and peers in the attempt to manage the demands of a task. Actually, to succeed in the social context of the classroom, teachers should help their students learn how to effectively use patterns of verbal and non-verbal communication, and how to interpret from others social cues that trigger emotional processes (Schultheiss and Köllner, 2014). Communication and social interaction also depend on the task at hand, being critical in dual or team activities. Team communication that occurs through both verbal and non-verbal behavior is usually deemed to be a fundamental component of coordinated performance (Eccles, 2010; Lausic et al., 2015) and less relevant in individual tasks.

To further assess the scale structure and to account for the first-order correlated dimensions, a higher order factor model and a nested-factor (bifactor) model were compared against the first-order factor model using CFA. The bifactor model, in particular, is contended to offer a number of key advantages including an easy interpretation of the general factor (which we named “affect/functionality” on the PBS-SPE scale) with direct influences on indicators, simultaneous examination of both general and specific influences on indicators, and identification of the particular contributions of the general and specific factors in predicting external criteria or variables (Reise, 2012; Canivez, 2016). However, both the higher order factor model and the bifactor model did not reach acceptable fit to the data, and therefore were not superior to the first-order factor model. These findings suggest that interpretation of pleasant/functional and unpleasant/dysfunctional factor scores can be straightforward, without a need to combine in some way the scores of the two factors.

### Measurement Invariance and Convergent/Nomological Validity

Additional aims of the study were to assess the invariance of the PBS-SPE scale on an independent sample, examine gender and age invariance, and investigate convergent and nomological validity. Results indicated a substantial measurement and structural invariance of the scale across the two study samples. MIMIC analysis and inspection of mean values of latent variables (Table 3) showed girls reporting lower scores of pleasant/functional states and higher scores of unpleasant/dysfunctional states than boys. Differences can derive from a lower participation of girls in spontaneous and organized sport and physical activities (García Bengoechea et al., 2010), as well as self-presentation issues, such as body image and physical perception (Labbrozzi et al., 2013). Furthermore, young girls and boys reported lower levels of both pleasant/functional and unpleasant/dysfunctional states compared to the older counterparts. This might reflect in youngsters a lower level of awareness of their own psychobiosocial states. Notwithstanding the differences in the mean scores of latent factors across gender and age, different item functioning was not observed when controlling for the underlying factor.

Results indicated that the latent factors of the PBS-SPE scale correlated with affective and motivation measures in the expected direction, thereby providing support for convergent and nomological validity of the PBS-SPE scale. In fact, the pleasant/functional factor of the PBS-SPE scale related positively to pleasant feelings, task-involving climate, intrinsic motivation, and identified regulation factors, and negatively to unpleasant feelings, external regulation, and amotivation factors. Moreover, the unpleasant/dysfunctional factor of the PBS-SPE scale related positively to unpleasant feelings and external regulation factors, and negatively to the intrinsic motivation factor. Similar relationships were found in previous studies showing that a task-involving climate improved students' pleasant/functional states and self-determined motivation (Bortoli et al., 2014, 2015, 2017). Interestingly, both correlation patterns of pleasant/functional states and unpleasant/dysfunctional states with the motivational variables are in line with the conceptualization of both the achievement goal theory (Treasure and Roberts, 1995) and self-determination theory (Ryan and Deci, 2017). The positive correlations observed between the psychobiosocial states index and the indices of the other summated scales (i.e., PACES, TIMCPEQ, and SIMS) also support the pattern of relationships. This is consistent with an integrative approach in physical education and sport that emphasizes an empowering task-involving, autonomy supportive, and socially supportive motivational climate, in contrast to a disempowering ego-involving and controlling motivational climate (Duda, 2013; Appleton and Duda, 2016).

### Limitations and Future Research Directions

A limitation of the current study was the small number of constructs used to assess the convergent and nomological validity of the PBS-SPE scale. Future research should further test the validity of the scale though measures of additional constructs (e.g., perceived competence, self-efficacy, enjoyment, and attitudes in physical activity and sport), as well as scale invariance across age, gender, different cultural groups, and participation in structured or unstructured physical and sport activities. It should also be noted that the scale was used in this study as a trait-like measure to gauge the student feelings usually experienced in physical education classes. A state-like version of the scale (i.e., “how you feel right now”) could provide useful information on current psychobiosocial states that can impact subsequent involvement in the activity and predict students' performance. The validity of the scale should be further tested in comparison with measures of emotional and motivational states, evaluations from others (e.g., teacher ratings), performance criteria, student level of current involvement in physical education tasks, and intention to engage in physical activity and sport outside of school (Di Battista et al., 2018).

A further limitation in this study is that we only examined the relationship between psychobiosocial states and four types of motivation lying in the autonomy–control continuum as construed in the organismic integration mini-theory (Ryan and Deci, 2017). The basic psychological needs theory is another mini-theory within the broad framework of the self-determination perspective that highlights the role of social and environmental support. This theory assumes that the three psychological needs of relatedness (being connected and accepted by significant others), competence (interacting effectively with the environment), and autonomy (having choice and freedom in action) typify self-determine motivation. These three key factors can integrate theoretical concepts stemming from achievement goal and self-determination theories, and influence students' psychobiosocial states.

Finally, the current investigation provides evidence of the validity of a measure to assess pleasant/functional and unpleasant/dysfunctional states as global dimensions. In this study we also gauged pleasant and unpleasant feelings as broad factors through the PACES. Within a dimensional approach, the Positive and Negative Affect Schedule (PANAS; Watson et al., 1988) is another widely used scale for the assessment of pleasant and unpleasant affect. Compared to the PACES and PANAS, the PBS-SPE scale is a more specific and comprehensive measure of psychobiosocial states occurring in physical education. However, more detailed information can derive taking a discrete approach through the assessment of the single interactive modalities that form a psychobiosocial state. Additional research in this direction is worth pursuing.

## Conclusion

Our study complements previous research on the assessment of psychobiosocial states in sport (Robazza et al., 2016; Ruiz et al., 2018), providing initial support for a measurement tool specific to the physical education context. According to several authors (e.g., Messick, 1995; Martinent et al., 2015), construct validation involves substantive, structural, and external features. In this study we have addressed all these requirements providing (a) a substantive theoretical rationale for investigating PBS-SPE, (b) evidence of factorial validity and reliability of the measure, and (c) evidence of convergent and nomological validity of the scale in the relationship with other variables consistent with a theoretical foundation.

Overall, our findings provide support for the PBS-SPE scale as a valid instrument in the assessment of a broad range of emotion-related psychobiosocial states occurring in the physical education context. It is therefore our contention that the use of the PBS-SPE scale will promote additional research on this relevant topic.

## Ethics Statement

Participants in the study and their parents or guardians signed an informed consent in accordance with the Declaration of Helsinki. Ethical approval for the study was obtained from the ethics committee for biomedical research of the G. d'Annunzio University of Chieti-Pescara, Italy, with anonymity and confidentiality being assured for all the participants.

## Author Contributions

All authors listed have made a substantial, direct and intellectual contribution to the work, and approved it for publication.

### Conflict of Interest Statement

The authors declare that the research was conducted in the absence of any commercial or financial relationships that could be construed as a potential conflict of interest.

## Appendix

**Appendix 1:** The psychobiosocial items of the PBS-SPE scale and the corresponding Italian translation.

**Items**	**Modality**
1. Enthusiastic, confident, carefree, joyful *Entusiasta, fiducioso, tranquillo, felice, gioioso*	Pleasant states(+)
2. (Fighting spirit, fierce, aggressive) *(Combattivo, grintoso, aggressivo)*	Anger(+)
3. Relaxed-, coordinated-, powerful-, effortless-movement *Movimento attivo, coordinato, dinamico, fluido*	Motor-behavioral(+)
4. Distracted, overloaded, doubtful, confused *Distratto, deconcentrato, dubbioso, confuso*	Cognitive(–)
5. Effective-, skillful-, reliable-, consistent-task execution *Prestazione efficace, abile, sicura, costante*	Operational(+)
6. (Uncommunicative, withdrawn, alone, disconnected) *(Chiuso, riservato, non-socievole, isolato)*	Communicative(–)
7. Nervous, restless, discontented, dissatisfied *Nervoso, irrequieto, scontento, insoddisfatto*	Anxiety(–)
8. Vigorous, energetic, physically-charged *Fisicamente vigoroso, pieno di energia, carico*	Bodily-somatic(+)
9. Sluggish, clumsy, uncoordinated, powerless-movement *Movimento debole, goffo, scoordinato, fiacco*	Motor-behavioral(–)
10. Alert, focused, attentive *Vigile, concentrato, attento*	Cognitive(+)
11. Unmotivated, uninterested, uncommitted *Demotivato, disinteressato, disimpegnato*	Motivational(–)
12. Overjoyed, complacent, pleased, satisfied *Allegro, compiaciuto, appagato, soddisfatto*	Pleasant states(+)
13. Ineffective-, unskillful-, unreliable-, inconsistent-task execution *Prestazione inefficace, scadente, incerta, instabile*	Operational(–)
14. (Communicative, outgoing, sociable, connected) *(Comunicativo, espansivo, socievole, cooperativo)*	Communicative(+)
15. Purposeful, determined, persistent, decisive *Risoluto, determinato, tenace, perseverante, deciso*	Volitional(+)
16. Worried, apprehensive, concerned, troubled *Preoccupato, angosciato, scoraggiato, turbato*	Anxiety(–)
17. Motivated, committed, inspired *Motivato, coinvolto, interessato*	Motivational(+)
18. Physically-tense, jittery, tired, exhausted *Fisicamente teso, nervoso, affaticato, esausto*	Bodily-somatic(–)
19. (Furious, resentful, irritated, annoyed) *(Furioso, risentito, rabbioso, astioso, irritato, infastidito)*	Anger(–)
20. Unwilling, undetermined, indecisive *Indeciso, incerto, esitante, rinunciatario, incostante*	Volitional(–)

*Items 2, 6, 14, 19 are in parentheses because they were not included in the final 16-item PBS-SPE scale. (+), item categorized as functional in the final version of the scale based on factor analyses; (–), item categorized as dysfunctional in the final version of the scale based on factor analyses*.
